# Measurement of Interfacial Adhesion Force with a 3D-Printed Fiber-Tip Microforce Sensor

**DOI:** 10.3390/bios12080629

**Published:** 2022-08-11

**Authors:** Mengqiang Zou, Changrui Liao, Yanping Chen, Zongsong Gan, Shen Liu, Dejun Liu, Li Liu, Yiping Wang

**Affiliations:** 1 Guangdong and Hong Kong Joint Research Centre for Optical Fibre Sensors, College of Physics and Optoelectronic Engineering, Shenzhen University, Shenzhen 518060, China; 2 Wuhan National Laboratory for Optoelectronics (WNLO), Huazhong University of Science and Technology (HUST), Wuhan 430074, China; 3 Department of Electronic Engineering, Chinese University of Hong Kong, Hong Kong, China

**Keywords:** femtosecond laser 3D printing, optical fiber sensor, fiber-tip microforce sensor (FTMS), clamped-beam probe, adhesion force measurement

## Abstract

With the current trend of device miniaturization, the measurement and control of interfacial adhesion forces are increasingly important in fields such as biomechanics and cell biology. However, conventional fiber optic force sensors with high Young’s modulus (>70 GPa) are usually unable to measure adhesion forces on the micro- or nano-Newton level on the surface of micro/nanoscale structures. Here, we demonstrate a method for interfacial adhesion force measurement in micro/nanoscale structures using a fiber-tip microforce sensor (FTMS). The FTMS, with microforce sensitivity of 1.05 nm/μN and force resolution of up to 19 nN, is fabricated using femtosecond laser two-photon polymerization nanolithography to program a clamped-beam probe on the end face of a single-mode fiber. As a typical verification test, the micronewton-level contact and noncontact adhesion forces on the surfaces of hydrogels were measured by FTMS. In addition, the noncontact adhesion of human hair was successfully measured with the sensor.

## 1. Introduction

Recently, along with the ongoing trend for device miniaturization, research on micro/nanoscale technology has also been conducted in depth. When the size of an object is reduced to the micro- or nanoscale, micro/nanoscale adhesion forces become extremely important because of the resulting increase in the surface area to volume ratio, and understanding of the adhesion behavior between surfaces has become an important topic [1,2,3]. Under general environmental conditions, the joint contributions of the capillary force, the electrostatic attractive force, hydrogen bonding and the van der Waals force constitutes the adhesion force [4,5,6]. Study of the adhesion forces and their molecular dynamics at interfaces is highly important in various fields, including materials science [7,8,9], biomedicine [10,11,12], aerospace [13], and micromechanics [14,15]. For example, adhesion can determine cell differentiation or enable a gecko to crawl easily on a smooth glass plate [16,17,18]. At present, the atomic force microscope (AFM) is the most commonly used instrument for measurement of interfacial interactions, and the AFM is often used to perform adhesion force measurements [19,20,21]. In 2017, Lai and Meng measured the adhesion force on hydrophilic silicon wafers using an AFM, and also investigated the effects of ambient humidity and the contact time on the adhesion force [22]. In 2020, Rokni and Wei used a novel atomic force nanomanipulation technique to measure the interlayer adhesion forces of both homogeneous and heterogeneous two-dimensional materials by attaching nanoscale two-dimensional crystal platforms to the tip of an AFM with high precision [14]. Despite the success of AFMs to date, their large size and complex operational procedures have limited the use of AFMs outside specialized laboratories [23,24]. Furthermore, conventional AFMs have remained largely unchanged over the past few decades in terms of force detection principles and the constituent materials of micro-cantilever beams, which greatly limit their measurement performance based on force sensing feedback. Therefore, there is an urgent need to develop a new generation of miniaturized, integrated, and flexible microscale force sensors to enable flexible measurement of the adhesion forces acting on micro/nanoscale structures. 

Optical fibers, which are compact, flexible, and resistant to electromagnetic interference, have become an all-optical platform for sensor miniaturization and integration [25,26,27,28]. However, there have been no known reports on the measurements of adhesion force that uses all-fiber microforce sensor, probably because the typical adhesion force values are on the micro- or even nanonewton level, which is too small to be detected when using fiber-optic force sensors composed of silica (Young’s modulus > 70 GPa) [29]. A common way to improve the sensitivity and force resolution of fiber optical microforce sensor is to reduce the diameter of fiber, such as making microfiber Bragg grating [16] or short sections of tapered microfibers. Femtosecond laser two-photon polymerization (TPP) nanolithography is a new 3D lithography technique that provides ultra-high machining accuracy with characteristic feature sizes of less than 100 nm. Ideally, any complex 3D polymer structure can be programmed using this process, and the formed polymer structure will have an ultra-low Young’s modulus [30]. Therefore, the combination of TPP nanolithography technology and the fiber optic platform offers the possibility of realization of high-resolution optical fiber microforce sensors.

**Figure 1:**
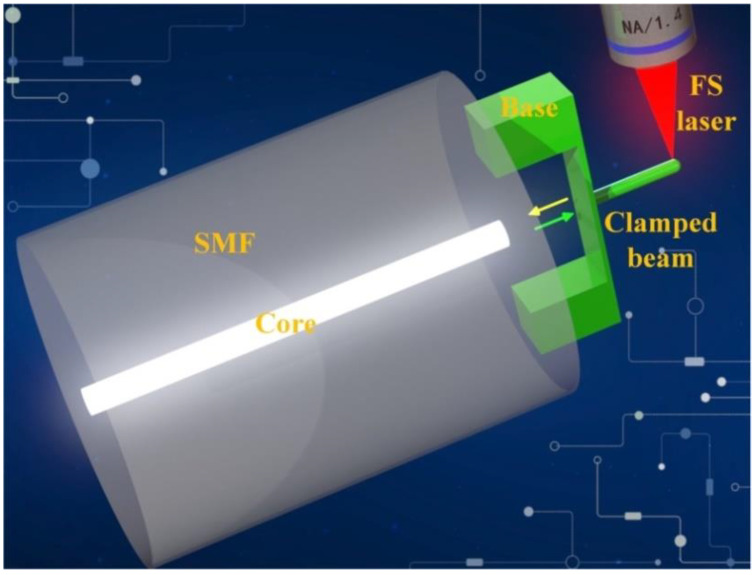
Schematic diagram of fiber-tip microforce sensor (FTMS).

In this paper, we demonstrate a fiber-tip microforce sensor (FTMS) that can be used to measure the μN-scale adhesion forces acting on the surfaces of micro/nanoscale structures. The FTMS was formed by using femtosecond laser TPP nanolithography to print a micron-sized clamped beam probe on the single-mode fiber (SMF) tip (Figure 1). A cylindrical probe is designed and micro-printed on the outer surface center of the clamped beam to pick up the external force. The optical fiber’s end face and the clamped beam formed a perfect Fabry-Perot microcavity. When the force is applied to the probe, i.e., the probe is pushed against a small object, the clamped beam will be deformed and the cavity length of the Fabry-Perot will be changed, resulting in a shift in the dip wavelength in the reflection spectrum of SMF. This dip wavelength achieved in the reflection spectrum was used to trace the weak vertical deformation of the clamped beam, and then the force on the probe was obtained. Microforce sensitivity of 1.05 nm/μN and a force sensing resolution of 19 nN were obtained. This force sensing resolution is an order of magnitude higher than that realized using a previously reported all-fiber force sensor based on a thin silica diaphragm (0.6 μN) [31]. The contact and noncontact adhesion forces of a hydrophilic hydrogel surface were measured successfully with this force sensor. The effect of the mass fraction of the hydrogel on the adhesion force was also studied, and a finite element analysis model of the adhesion between the probe and the hydrogel surface was established. The contributions from both the capillary force and the electrostatic attractive force to the adhesion force have been analyzed in detail. The measurement results of adhesion force provide mechanical reference for hydrogel based biological scaffold or cell adhesion. In addition, the non-contact adhesion force of adult female hair was measured by FTMS. Therefore, we believe that FTMS will be a beneficial tool for adhesion force measurement of bio-relevant materials. Furthermore, the FTMS provides a simple and flexible method for quantitative measurement of the adhesion of micro/nanoscale structure surfaces, and may also provide a new interface force test method that cannot be performed using current commercial AFMs.

## 2. Materials and Methods

### 2.1. Sensing Principle and Fabrication

**Figure 2:**
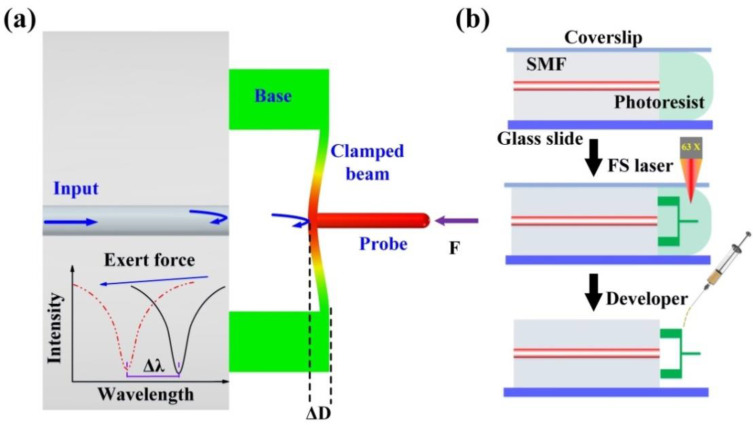
Sensing schematic diagram of the proposed sensor. (**a**) Simulation results for the device under an applied external force, (**b**) FTMS fabrication process.

Figure 2a illustrates a sensing schematic diagram of the proposed FTMS. Three important components, including a cylindrical probe, a clamped beam, and a pair of bases, were microprinted directly onto the end facet of an SMF using the TPP microprinting technique. The cylindrical probe is on the outer surface center and is perpendicular to the clamped beam. This clamped beam is parallel to the fiber end face and is supported by the printed two bases. Thus, light will be reflected back to the fiber at both the fiber end facet and the clamped beam, forming interference fringes in the fiber. This interference fringes will vary as the distance between the clamped beam and the fiber end face changes. This distance, which we refer to as the cavity length of the Fabry-Perot cavity defined by inner surface of the clamped beam and the fiber end face, is easily affected by external force. When an external force acts on the probe, the clamped beam will be deformed, thus reducing the cavity length. This cavity length reduction (Δ*L*) leads to a dip wavelength shift (Δ*λ*) in the reflection spectrum, as illustrated in the inset in Figure 2a. The wavelength shifts linearly with the cavity length change as follows [32]:

(1) $$\frac{\Delta \lambda }{\Delta L}=\frac{\lambda }{L}$$

where *λ* and *L* are the traced dip wavelength of the reflection spectrum and the cavity length, respectively. Therefore, the deflection induced by the external force can be obtained through demodulating the wavelength shift directly. It should be noted that the clamped beam has a nonnegligible thickness, and three Fabry-Perot interferometers (FPIs) are formed by three reflective mirrors, i.e., the fiber end face, the inner and outer surfaces of the clamped beam. Only the air FPI is selected for demodulation. The detailed TPP microprinting process has three steps, as shown in Figure 2b. 

First, an SMF (SMF-28, Corning Inc., New York, NY, USA) with a well-cleaved end facet was placed on a well-cleaned glass slide. Then, a droplet of photoresist was dropped onto the glass slide to cover the entire end facet of the SMF. A coverslip was placed onto the SMF upper surface to stop the photoresist from flowing away, as shown in the top part of Figure 2b.

Second, the glass slide described above was then mounted on a 3D fabrication strategy for structure microprinting, as shown in the middle part of Figure 2b. An oil immersion objective lens (63×, numerical aperture (NA) of 1.4) was used to help the laser beam to focus on the fiber tip. In the microprinting process, a commercial femtosecond laser (fs) was used. The repetition frequency and the central wavelength of the fs laser are 200 kHz and 1026 nm, respectively. To improve the fabrication efficiency, the output power of the fs laser and the scanning velocity were set as 1.8 mW and 600 μm/s, respectively. The laser printing procedure begins inside the fiber to enhance the adhesion between the printed structure and the fiber end face. Herein, the printed bases have a length of 20 μm, a width of 20 μm, and a height of 30 μm; the clamped beam has a length of 100 μm, a width of 20 μm, and a height of 3 μm; and the probe has a diameter of 5 μm and a length of 35 μm. 

Finally, as shown in the lower part of Figure 2b, two drops of an acetone and isopropanol mixture (volume ratio of 1:5) were added carefully to the fiber tip to clean off any uncured photoresist. This process may be repeated if necessary, and after this step, the structure was successfully micro-printed on the fiber end facet.

### 2.2. Characterization

**Figure 3:**
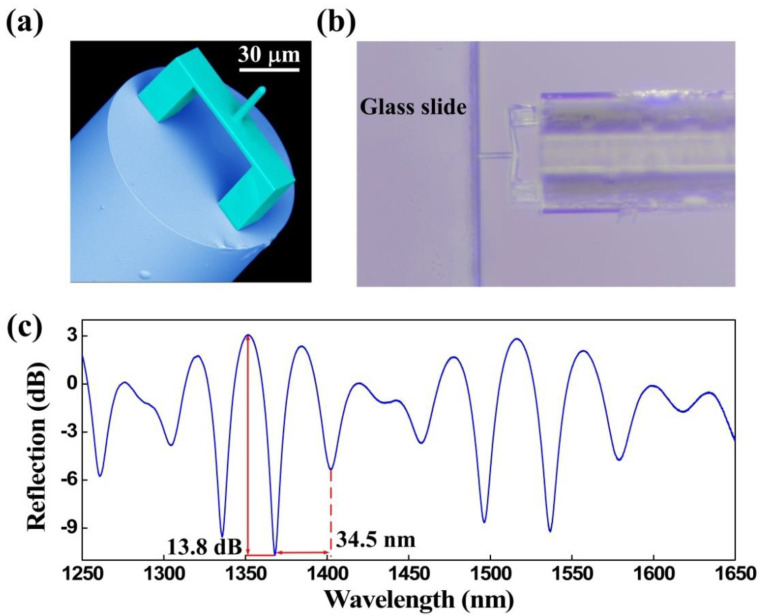
Characteristics of the FTMS. (**a**) SEM image of the FTMS. (**b**) Optical microscope image of the FTMS when pressed on the glass slide edge. (**c**) Reflection spectrum for the FTMS.

Three types of characterization analysis were performed for the proposed FTMS, namely, structural morphology, elastic properties, and optical properties. A scanning electron microscope (SEM, Phenom Pro) was used to photograph the morphology of FTMS, employing a voltage of 5 kV [30]. Figure 3a presents a SEM image of the fs micro-printed structure. The image clearly shows that the printed structure was printed as designed, i.e., the clamped beam with the cylindrical probe on the outer surface center lies parallel to the fiber end facet. Furthermore, this polymerized structure has smoothed surfaces that help to improve the contrast of the reflection spectrum. Leica Microsystems microscope (DM 27000M RL/TL) was used to obtain the elastic properties that can reflect the deformation recovery of FTMS [28]. The polymerized structure is shown to have good elastic properties by simply pressing it on the glass slide edge. As Figure 3b shows, the clamped beam deforms when it is pressed into the glass slide edge, but it recovers when released. The optical properties of FTMS were measured using a broadband light source (BBS, 600–1700 nm, from Fiber Lake Co., Ltd., Shenzhen, China), a 3-dB fiber coupler, and an optical spectrum analyzer (OSA with resolution of 0.02 nm, YOKOGAWA, AQ6317C) [30]. Figure 3c shows the corresponding reflection spectrum of the polymer structure. The polymer structure has a free spectral range (FSR) of ~34.5 nm and an extinction ratio of ~13.8 dB. This large extinction ratio, along with the elastic properties of the polymer structure, lays a foundation that makes it possible to measure microforces.

## 3. Results

### 3.1. Force Measurement

**Figure 4:**
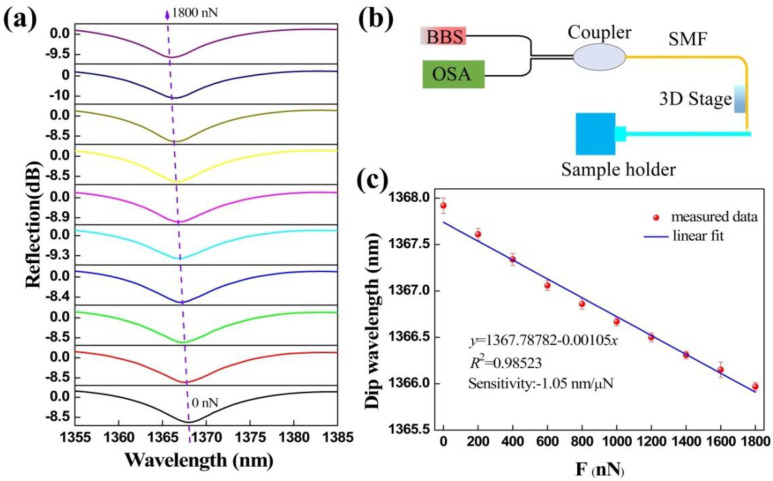
Microforce measurements. (**a**) Reflection spectral evolution of the FTMS while the external force increases from 0 to 1800 nN. (**b**) Measurement system setup. (**c**) Linear relationship between dip wavelength and microforce. The line is the linear fitting of measured data points and the error bar is obtained by critically repeating the experiment of force measurement three times.

Before the adhesion force acting on the surfaces of micro/nanoscale structures was measured, the microforce sensitivity of the proposed device were measured through the experimental setup shown in Figure 4b. Microforce sensitivity can be obtained as the drift of dip wavelength caused by FTMS under the action of unit force [30]. The experimental setup consists of a BBS, an OSA, a 3-dB coupler, and a 3D electric translation stage that can micro operate the sensor. A segment of coating-stripped SMF with a certain length is fixed on the sample holder in the form of a cantilever and perpendicular to the sensor’s clamped beam probe. The sensor probe was pushed against the free end of the SMF and the SMF deflected. The reaction force on the sensor probe can be derived from the following equation

*F* = 3*EI*Δ*L*/*L*^3^, (2)

where *L* is the length of the SMF, Δ*L* is the deflection of the free end of the SMF, *E* is the Young’s modulus of the SMF, and *I* is the circular second moment of area of the SMF that can be calculated from the diameter of the SMF. Therefore, similar to the previously reported literature [30,31] we can get the drift of FTMS reflection spectra under different external forces, and then obtain its microforce sensitivity.

The responses of the FTMS reflection spectra to different applied microforces are shown in Figure 4a. The reflection spectrum has a blue wavelength shift as the microforce applied to the FTMS increases. A total drift wavelength of approximately 1.8 nm was obtained when the microforce was increased from 0 to 1800 nN in increments of 200 nN. These experimental results agree well with the theoretical analysis. Because of the applied external force, the cavity length of the Fabry-Perot microcavity decreases, causing a reduction in the optical path length. Excitingly, the extinction ratio and the full width at half maximum (FWHM) of the reflection spectrum both remain basically unchanged. The FTMS measures the microforce by monitoring the dip wavelength shift indicated by the purple dotted arrow in the figure. These dip wavelengths were extracted and subjected to a linear fitting. The relationship obtained between the dip wavelength and the microforce is illustrated in Figure 4c. Through linear fitting, the microforce sensitivity of the FTMS was calculated to be 1.05 nm/μN with an R-squared (R^2^) value of 0.98523, indicating the good linearity of the wavelength and microforce responses. Through this process, we determined the functional relationship between the microforce and the dip wavelength. The force sensing resolution can be obtained by the ratio of the resolution of the optical spectrum analyzer that used to the microforce sensitivity. The resolution of the FTMS is 19 nN within the limited 0.02 nm resolution of the optical spectrum analyzer (OSA, YOKOGAWA, AQ6317C) [33], which is an order of magnitude better than that of previously reported all-fiber force sensors that were based on a thin silica diaphragm [31]. Experimentally, the maximum force that can be measured using this device was determined to be 2.5 mN. 

### 3.2. Interface Adhesion Force Measurement

Agarose hydrogel, a type of gel material that uses water as its dispersion medium, has strong hydrophilicity, and it is easy to adsorb a multilayer water film on its surface to produce a strong adhesion force [34]. Because of their good biocompatibility, controlled mechanical properties, and ability to promote cell attachment and proliferation, hydrogels are used widely in biological scaffolds, biosensor, cell cultures, drug delivery, and regenerative medicine. Therefore, study of the mechanical properties of hydrogels, e.g., their hardness and adhesion, is highly significant for construction of biofunctional tissues [35]. To prepare the agarose hydrogels required, the agarose powder matrix and deionized water were first mixed at a specific mass percentage; then, the temperature of the mixture was increased to 90 °C and kept at that temperature for 20 min, before finally being cooled down to room temperature to form the desired hydrogels. Two agarose hydrogels with mass fractions of 1.8% and 0.6% were prepared, and all treatments were performed in an ultra-clean room.

**Figure 5:**
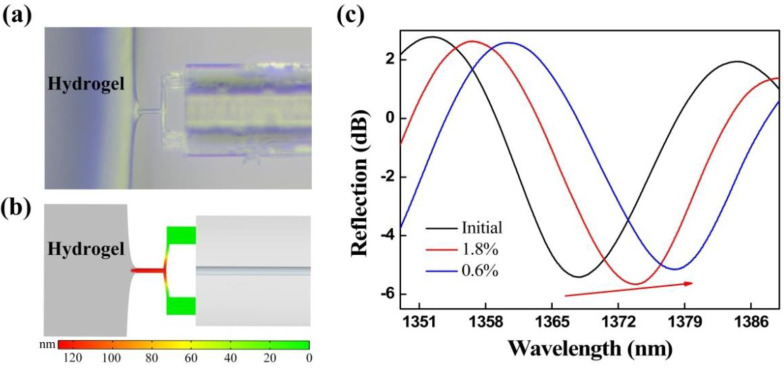
(**a**) Probe of the FTMS was encapsulated by micro-protrusions of the hydrogel under the effect of the adhesion force. (**b**) Simulation results for adhesion of the FTMS to agarose hydrogels. (**c**) Evolution of the reflection spectrum under the action of the adhesion.

The FTMS, driven by a 3D electric translation stage, gradually moved toward the fixed agarose hydrogel. Agarose hydrogel used in the experiment is a kind of highly hydrophilic three-dimensional reticular gel, which relies on secondary chains between sugar chains such as hydrogen bonds to maintain the reticular structure, so as to maintain a certain shape and volume [36]. Due to the existence of cross-linking network, hydrogels can swell and retain a large amount of water [37]. The absorption of water is closely related to the degree of cross-linking. The higher the degree of cross-linking, the lower the amount of water absorption, which is similar to soft tissue. The mechanical properties of hydrogels can be tuned and modified by crosslinking concentration and additives. Furthermore, due to their considerable water content, hydrogels also possess a degree of flexibility analogous to that of natural tissue [38]. The adhesion force worked when the probe was close to the edge of the agarose hydrogel (<1 μm), i.e., the mutual attraction between the probe and the agarose hydrogel worked to generate micro-protrusions in the hydrogel, and the probe was then encapsulated by these micro-protrusions, as shown in Figure 5a. The probe was then gradually pulled away from the micro-protrusions, and the maximum pull-off force, i.e., when the probe was separated from the agarose hydrogel, is the adhesion force. Here, the adhesion force is mainly composed of the capillary force and the van der Waals force. We established an adhesion force model of the FTMS and the agarose hydrogel was using COMSOL Multiphysics^®^ software, as shown in Figure 5b. The simulation results are similar to the experimental phenomena, i.e., the micro-protrusions are produced on the hydrogel surface because of the adhesive force, and the clamped-beam probe of the FTMS was stretched and deformed by the adhesion force, thus increasing the Fabry-Perot cavity length. Figure 5c presents the reflection spectra evolution of the sensor that occurred when the probe was pulled off the two different agarose hydrogels with mass fractions of 1.8% and 0.6%. The initial reflection spectrum and the reflection spectra upon pull-off from the two different agarose hydrogels are shown as the black solid, red solid, and blue solid lines, respectively. The wavelength shows a red shift, indicating that the Fabry-Perot cavity length increases because of the adhesive force. The adhesion forces of the agarose hydrogels with mass fractions of 1.8% and 0.6% were calculated to be 5.86 μN and 9.77 μN, respectively. Hydrogels with the mass fraction of 0.6% contain more water and thus tend to produce a thicker water film on the surface. Consequently, a larger effective contact area between the probe and the hydrogel and an enhanced capillary force were obtained, thus producing a greater adhesion force. In addition, the FTMS can measure noncontact adhesion in addition to contact adhesion. 

**Figure 6:**
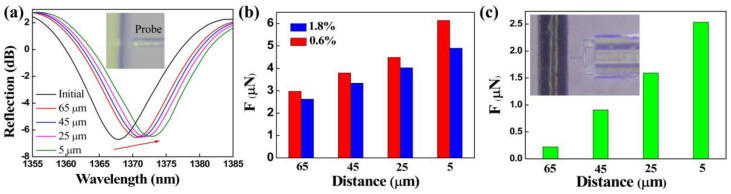
(**a**) Drift of the FTMS reflection spectrum under the action of the noncontact adhesion force of hydrogels. (**b**) Noncontact adhesion forces between the probe and the agarose hydrogels at different distances. (**c**) Noncontact adhesion forces measurement of human hair.

As the inset of Figure 6a shows, the clamped-beam probe of the FTMS is close to but not in contact with the 1.8% mass fraction agarose hydrogel. There are no micro-protrusions at the edge of the hydrogel. However, there is still an adhesion force acting between the probe and the hydrogel surface that can be attributed to long-range electrostatic attraction. Different noncontact adhesion forces, i.e., at different distances between the probe and the edge of the gel, were measured. Figure 6a presents the evolution in the FTMS spectra as the distance between the probe and the edge of the gel decreases from 65 μm to 5 μm. The dip wavelength shows a red shift as the distance decreases gradually, thus indicating an increase in the noncontact adhesion force. Figure 6b shows the noncontact adhesion forces of the agarose hydrogels with mass fractions of 1.8% and 0.6% at different distances. The noncontact adhesion force of each agarose hydrogel increases as the distance decreases, and the noncontact adhesion forces were calculated to be 4.89 μN and 6.14 μN for the agarose hydrogels with mass fractions of 1.8% and 0.6%, respectively, at a distance of 5 μm. These results clearly show that the gel adhesive with the mass fraction of 0.6% has a greater noncontact adhesion force at the same distance in each case. Similarly, the noncontact adhesion force between the probe and human hair (black hair from Chinese adult women) at different distances was measured by FTMS, as shown in Figure 6c. It can be seen that the adhesion force decreases rapidly with the increase of distance. The noncontact adhesion force is only 221 nN when the distance reaches 65 μm.

For hydrogels and human hair on the micro scale, the surface effect is greatly enhanced due to the increase of the ratio of surface area to volume, and the surface effect will be better than the inertia effect, which makes the adhesion force become extremely important [1,4,39]. Under general environmental conditions, adhesion force has many sources, including van der Waals force, electrostatic attractive force, hydrogen bonding force, capillary force, and other interaction forces generated by the physics and chemistry of the interaction surface [6]. The contact adhesion of agarose aqueous gel is mainly dominated by capillary force and van der Waals force. Water is adsorbed from the surrounding environment to the surface of the hydrogel to form a water film. The condensation of water leads to high interfacial force, and with the increase of water film, the capillary force increases, and the adhesion force will increase significantly [40]. The non-contact adhesion force in the experiment belongs to long-range force, in which electrostatic attractive force plays a leading role. Therefore, this non-contact adhesion force can be attributed to long-range electrostatic attraction [1].

AFM is the most commonly used tool to measure the interfacial adhesion force of micro/nanoscale structures. AFM relies on the cantilever probe installed on it to separate after contacting with the surface of the sample to be tested. In the process of separation, the cantilever will be deflected under the action of adhesion force. The adhesion force between the cantilever probe and the micro/nanoscale structure can be determined by monitoring the deflection of the cantilever. Similar to the AFM, the clamped beam probe of the proposed FTMS is separated after contacting the surface of the sample to be measured. This process will cause the deformation of the clamped beam, resulting in the drift of the FTMS reflection spectrum, and the value of adhesion force can be obtained from the drift of the reflection spectrum. Finally, it can provide a simple, efficient, and accurate adhesion force measurement method like AFM.

## 4. Conclusions

In conclusion, we have provided an alternative method to enable quantitative determination of the adhesion forces of micro/nanoscale structures using a fiber-tip polymer microforce sensor (FTMS). This FTMS is fabricated by 3D printing of a clamped-beam probe on the end face of an SMF using femtosecond laser TPP nanolithography. The resulting FTMS has microforce sensitivity of 1.05 nm/μN and force resolution of 19 nN. This FTMS can be used to measure both positive and negative forces. The contact and noncontact adhesion forces of agarose hydrogel were measured successfully using the proposed sensor, and the influence of the hydrogel’s mass fraction on the adhesion force was also studied. The contact adhesion behavior of the agarose hydrogel was simulated by finite element analysis. The effects of the capillary force and the electrostatic attraction force on the adhesion of the hydrogel were studied. In addition, the adhesion force of human hair has been successfully measured using this sensor. The FTMS has great application prospects in the measurement of adhesion of biological samples because of its compact size, usage flexibility, all-optical operation, and biocompatibility. To the best of our knowledge, this is the first report of the use of a miniature all-fiber microforce sensor to measure adhesion forces on the micronewton level for micro/nanoscale structures, and this approach will provide a useful addition to the existing AFM adhesion force measurement techniques.

## Data Availability

The experimental data is contained within the article.
